# Do Variants Associated with Susceptibility to Pancreatic Cancer and Type 2 Diabetes Reciprocally Affect Risk?

**DOI:** 10.1371/journal.pone.0117230

**Published:** 2015-02-06

**Authors:** Lang Wu, Kari G. Rabe, Gloria M. Petersen

**Affiliations:** 1 Department of Health Sciences Research, Mayo Clinic, Rochester, Minnesota, United States of America; 2 Center for Clinical and Translational Science; Mayo Clinic, Rochester, Minnesota, United States of America; MD Anderson Cancer Center, UNITED STATES

## Abstract

**Objectives:**

Although type 2 diabetes mellitus is a known risk factor for pancreatic cancer, the existence of shared genetic susceptibility is largely unknown. We evaluated whether any reported genetic risk variants of either disease found by genome-wide association studies reciprocally confer susceptibility.

**Methods:**

Data that were generated in previous genome-wide association studies (GENEVA Type 2 Diabetes; PanScan) were obtained through the National Institutes of Health database of Genotypes and Phenotypes (dbGaP). Using the PanScan datasets, we tested for association of 38 variants within 37 genomic regions known to be susceptibility factors for type 2 diabetes. We further examined whether type 2 diabetes variants predispose to pancreatic cancer risk stratified by diabetes status. Correspondingly, we examined the association of fourteen pancreatic cancer susceptibility variants within eight genomic regions in the GENEVA Type 2 Diabetes dataset.

**Results:**

Four plausible associations of diabetes variants and pancreatic cancer risk were detected at a significance threshold of p = 0.05, and one pancreatic cancer susceptibility variant was associated with diabetes risk at threshold of p = 0.05, but none remained significant after correction for multiple comparisons.

**Conclusion:**

Currently identified GWAS susceptibility variants are unlikely to explain the potential shared genetic etiology between Type 2 diabetes and pancreatic cancer.

## Introduction

It has been long recognized that Type 2 diabetes mellitus (T2DM) is a risk factor for development of pancreatic cancer (PaC). A meta-analysis of 17 case-control and 19 cohort or nested case-control studies found that the combined odds ratio (OR) was 1.82 for PaC risk among diabetic subjects compared to nondiabetic subjects [1]. Another meta-analysis supported a conclusion that long-standing diabetes of at least 5 years duration has a RR of 2.0 (95% CI, 1.2 to 3.2) for development of PaC [2]. Recently, a study analyzing 15 case-control studies from the Pancreatic Cancer Case-Control Consortium (PanC4) found that the OR for a diagnosis of diabetes two or more years before the diagnosis of PaC (long-standing diabetes) is 1.90 (95% CI:1.72 to 2.09), and such an association even persists for 20 or more years after diabetes was diagnosed (OR = 1.30, 95% CI 1.17 to 2.03) [3]. Thus, longstanding diabetes can increase risk of developing PaC. Interestingly, a population-based case-control study found that first-degree relative history of T2DM is associated with increased risk of PaC (OR 1.95, 95% CI 1.23–3.09) [4]. Any underlying link to explain these observations is unknown. Given the observations regarding family history, one potential explanation is that there is a shared genetic etiology for risk of both T2DM and PaC. With the significant research investment in genetic analysis of both diseases, particularly in genome-wide association studies (GWAS), it is now possible to test whether reported genetic susceptibility variants might be reciprocally associated with risk of developing either condition.

GWAS is a design that aims to agnostically identify genetic susceptibility to complex diseases[5]. This study design has been extensively employed in T2DM and PaC, and many genetic susceptibility variants have been identified [6,7]. For example, the GENEVA Genes and Environment Initiatives in Type 2 Diabetes study, which includes the Nurses' Health Study (NHS) and the Health Professionals Follow-up Study (HPFS) subsets, contributed to understanding the genetic predisposition to T2DM [8]. PanScan studies, comprised of PanScan 1, PanScan 2, and PanScan 3, have reported eight susceptibility loci for PaC [9,10,11].

**Figure 1:**
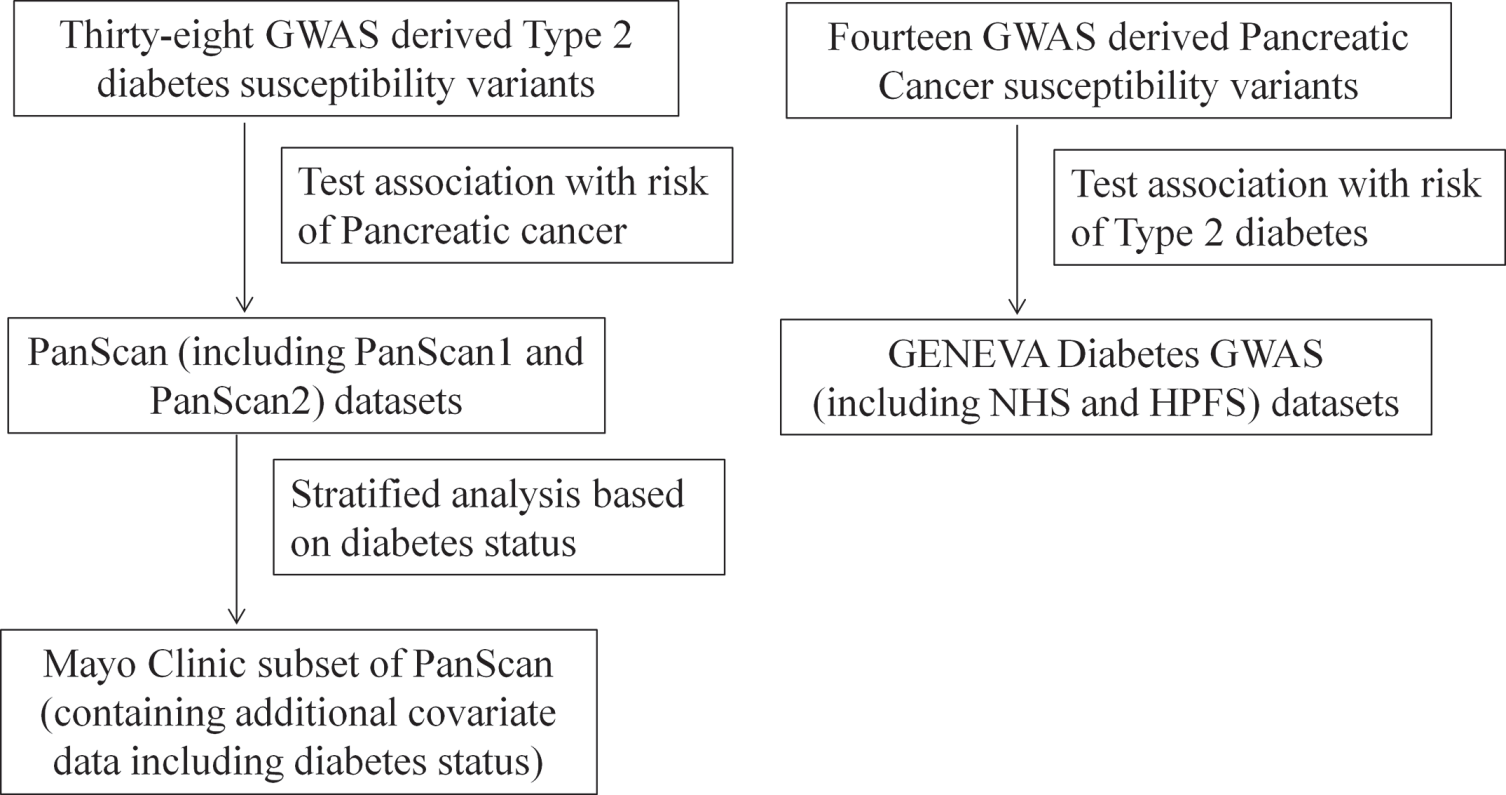
Study Design. Distinct comparisons examined T2DM SNPs in PanScan datasets and examined PaC SNPs in the GENEVA T2DM dataset.

We hypothesized that the susceptibility variants identified though these published GWAS contribute to a shared etiology. A study examining the association between T2DM susceptibility variants and PaC risk using the PanScan 1 dataset reported three variants that were associated with PaC risk [12]. However, this study did not validate their findings using an independent dataset. Associations between PaC susceptibility variants and T2DM risk were not estimated, thus limiting the knowledge on the potential role of these GWAS-derived variants in any shared etiology. To systematically evaluate this question, we analyzed the combined PanScan 1and 2 data to evaluate whether T2DM susceptibility variants can predispose to PaC risk. Additionally, since around 15% of pancreatic cancer patients have a diagnosis of diabetes two or more years before the cancer diagnosis [3], it was of interest to assess whether the association between T2DM susceptibility variants and PaC risk can be differentiated by diabetes status of the studied individuals. We thus conducted a stratified analysis based on diabetes status (contrasting long-standing diabetes and no history of diabetes). We then used the GENEVA Type 2 Diabetes datasets to evaluate whether reported PaC susceptibility variants are associated with risk of T2DM. The overall study design is illustrated in Fig. 1.

## Materials and Methods

### Genotype data

Two publicly available GWAS datasets of GENEVA Type 2 Diabetes and PanScan were obtained through dbGaP [13] to investigate our hypothesis.

PanScan included genotypes generated in two phases: PanScan 1 and PanScan 2 (http://www.ncbi.nlm.nih.gov/projects/gap/cgi-bin/study.cgi?study_id=phs000206.v3.p2). Briefly, PanScan 1 consisted of 12 prospective cohort studies and a Mayo Clinic case-control study. The Illumina HumanHap550v3.0 genotyping platform genotyped 558,542 SNPs. Genotyping in PanScan 2 augmented PanScan 1 with 8 case-control studies from the PanC4. The Illumina Human610_Quadv1_B genotyping platform genotyped 581,188 SNPs. Age, sex, study site, and race comprised available covariate data. The Mayo Clinic subset drawn from PanScan 1 and 2 comprised 654 cases and 618 controls, with additional covariate information available: smoking status, first degree family history of cancer, body mass index (BMI), and diabetes status [14].

The GENEVA Genes and Environment Initiatives in Type 2 Diabetes study consisted of GWAS data generated using the NHS and HPFS cohorts. This dataset (http://www.ncbi.nlm.nih.gov/projects/gap/cgi-bin/study.cgi?study_id=phs000091.v2.p1) included two subsets: NHS (1581 cases and 1810 controls), and HPFS (1164 T2DM cases and 1338 controls, and 68 cases of uncertain diabetes type). Both studies used the Affymetrix Affy_6.0 genotyping platform and 934,940 SNPs were genotyped. Available covariates included family history of diabetes, high blood pressure, high blood cholesterol, smoking status, physical activity, age, BMI, alcohol intake, fat intake measures, magnesium intake, cereal fiber intake, heme iron intake, glycemic load, and gender.

### Quality Control filters

PanScan genotype data had been filtered previously for call rate, relatedness, controls that became cases, and sex chromosome abnormalities. If duplicates were genotyped, the individual with the higher call rate was used. The final analysis included 3360 cases and 3468 controls in the combined PanScan data. The Mayo Clinic subset analysis consisted of 63 cases and 23 controls in the long-standing diabetes group (a diabetes diagnosis of 2 or more years earlier than diagnosis of pancreatic cancer for cases, or 2 or more years earlier than date of enrollment for controls), and 448 cases and 557 controls without diabetes.

The GENEVA datasets had been pre-filtered for sex chromosome abnormalities, sample identity, and call rate. We further filtered for relatedness of the sample and Hardy–Weinberg equilibrium (HWE) for variants studied in both NHS and HPFS sets. For the HPFS, we additionally filtered out subjects with uncertain diabetes status. The final sample for our analysis consisted of 1579 cases and 1801 controls from the NHS set (all females), 1162 cases and 1336 controls from the HPFS set (all males), for a total of 2741 cases and 3137 controls.

### Genetic Variant Selection

Thirty-eight T2DM susceptibility SNPs [8,15,16,17,18,19,20,21,22,23,24,25] and 14 PaC susceptibility SNPs [9,10,11] from published GWAS were included in our analyses. Thirty-seven of the 38 T2DM predisposition variants and five of the 14 PaC predisposition variants had been genotyped and were available in the PanScan datasets and GENEVA T2DM datasets, respectively. To represent variants which were not captured in the GWAS data, we captured the genotype by identifying SNPs in high LD (r^2^>0.5) as determined by Haploview [26]. If multiple variants were identified for a variant of interest not in the GWAS, the SNP with the smallest p-value was chosen as representative for the association.

### Statistical Analysis

Unconditional multivariable logistic regression assuming an additive model was employed in the association analyses. Available covariates were adjusted in the analyses. For associations reaching p = 0.05 threshold, a Bonferroni correction was conducted to adjust for multiple comparisons. Specifically, for the PaC dataset analysis, a threshold of 0.05/37 = 0.0014 was used since all 38 variants from 37 genomic regions were tested; for the T2DM dataset analysis, a threshold of 0.05/6 = 0.008 was used since 11 variants from 6 genomic loci were tested. Additionally, a correction of false discovery rate (FDR) was conducted to evaluate the significance [27].

For the PanScan data, covariates in the model included age, sex, study site, genotypic race (Eigenstrat principal components 1 and 2), and other significant principal components. We further conducted a stratified analysis in the Mayo Clinic subset according to diabetes status to investigate whether the T2DM susceptibility variants have a specific effect on either (1) long-standing diabetic PaC patients (cases who had long-standing diabetes 2 or more years before the diagnosis of pancreatic cancer) versus long-standing diabetic PaC controls, or (2) PaC cases and controls without diabetes.

In the GENEVA Type 2 Diabetes datasets, we conducted similar adjusted analysis. Covariates in the model included age, principal components (the first two components were used for NHS and 20 components were used for HPFS), family history of diabetes, high blood pressure, high blood cholesterol, smoking status, physical activity, BMI, alcohol intake, fat intake measures, magnesium intake, cereal fiber intake, heme iron intake, and glycemic load. As the NHS and HPFS set each contained a single gender, we did not include gender as a covariate in analyses of the separate sets but included gender in the combined set.

In all analyses, odds ratio (OR) and 95% confidence intervals (CI) were computed using Plink (http://pngu.mgh.harvard.edu/purcell/plink/) [28].

## Results

### T2DM SNPs and pancreatic cancer risk in the PanScan datasets

All 38 T2DM susceptibility variants that had been reported in the literature were included in the analyses as either observed in the GWAS data (n = 37) or represented by SNPs in high LD (n = 1). The results from the combined PanScan datasets are shown in Table 1.

**Table 1:** Association between Type 2 diabetes (T2DM) susceptibility variants and pancreatic cancer risk.

Chromosome	SNP	Gene Region	T2DM odds ratio (OR)	Risk/non-risk allele Risk allele frequency*	Combined PanScan 1 & 2^~^ (3360 cases and 3468 controls)	
					Odds Ratio (95% C.I.)	p
**SNPS observed in GWAS**						
1	rs2641348	NOTCH2	1.13	C/T	1.03	0.58
				0.11	(0.92–1.15)	
1	rs340874	PROX1	1.07	G/A	1.00	0.96
				0.54	(0.93–1.07)	
2	rs13414140	THADA	1.15	C/T	0.98	0.67
				0.91	(0.88–1.09)	
2	rs243021	BCL11A	1.08	T/C	0.90	0.0047
				0.46	(0.84–0.97)	
2	rs2943641	IRS1	1.11	C/T	1.00	0.998
				0.63	(0.93–1.08)	
2	rs780094	GCKR	1.06	G/A	0.99	0.69
				0.59	(0.92–1.06)	
3	rs2877716	ADCY5	1.12	C/T	1.08	0.06
				0.81	(1.0–1.17)	
3	rs4402960	IGF2BP2	1.14	T/G	1.03	0.43
				0.29	(0.96–1.11)	
3	rs4411878	ADAMTS9	1.08	C/T	1.03	0.42
				0.79	(0.95–1.12)	
3	rs6802898	PPARG	1.14	C/T	1.06	0.30
				0.88	(0.95–1.17)	
4	rs10012946	WFS1	1.12	C/T	1.00	0.89
				0.63	(0.93–1.07)	
5	rs7708285	ZEBD3	1.08	G/A	1.05	0.26
				0.31	(0.97–1.13)	
6	rs7756992	CDKAL1	1.14	G/A	0.96	0.32
				0.27	(0.89–1.04)	
7	rs13234407	KLF14	1.07	G/A	1.05	0.14
				0.54	(0.98–1.13)	
7	rs1635852	JAZF1	1.1	T/C	0.90	0.003
				0.46	(0.84–0.96)	
7	rs2191348	DGKB-TMEM195	1.06	T/G	1.00	0.97
				0.52	(0.93–1.07)	
7	rs4607517	GCK	1.07	A/G	1.11	0.03
				0.18	(1.01–1.21)	
8	rs13266634	SLC30A8	1.15	C/T	1.02	0.61
				0.71	(0.95–1.10)	
8	rs896854	TP53IMP1	1.06	A/G	1.02	0.67
				0.46	(0.95–1.09)	
9	rs10512085	CHCHD9	1.11	A/G	1.10	0. 16
				0.92	(0.96–1.27)	
9	rs2383208	CDKN2A/B	1.20	A/G	0.99	0.83
				0.83	(0.90–1.08)	
10	rs1111875	HHEX/IDE	1.15	G/A	0.97	0.42
				0.55	(0.90–1.04)	
10	rs11257655	CDC123	1.11	T/C	1.06	0.16
				0.24	(0.98–1.16)	
10	rs7903146	TCF7L2	1.37	T/C	1.01	0.84
				0.31	(0.93–1.09)	
11	rs1387153	MTNR1B	1.15	T/C	1.05	0.26
				0.28	(0.97–1.13)	
11	rs1552224	CENTD2	1.14	T/G	1.03	0.56
				0.84	(0.93–1.14)	
11	rs2237892	KCNQ1	1.29	C/T	1.04	0.58
				0.93	(0.90–1.20)	
11	rs2334499	H19/IGF2	1.08	T/C	1.03	0.46
				0.43	(0.96–1.10)	
11	rs5215	KCNJ11	1.14	C/T	0.97	0.47
				0.34	(0.91–1.05)	
12	rs1353362	TSPAN8	1.09	C/T	0.98	0.52
				0.29	(0.90–1.05)	
12	rs2612067	HMGA2	1.10	C/A	1.04	0.54
				0.12	(0.93–1.16)	
12	rs7965349	HNF1A	1.07	C/T	0.95	0.27
				0.81	(0.87–1.04)	
15	rs4778582	ZFAND6	1.06	A/G	1.01	0.85
				0.67	(0.94–1.08)	
15	rs8042680	PRC1	1.07	A/C	0.99	0.81
				0.34	(0.92–1.07)	
16	rs8050136	FTO	1.17	A/C	1.08	0.03
				0.42	(1.01–1.16)	
17	rs4430796	HNF1B	1.10	G/A	1.05	0.21
				0. 47	(0.98–1.12)	
X	rs5945326^f^	DUSP9	1.27	A/G	1.01	0.90
				0.74	(0.89–1.13)	
X	rs5945326^m^	DUSP9	1.27	A/G	0.93	0.35
				0.74	(0.79–1.09)	
**SNPs in high LD with susceptibility variants**						
11	rs231362	KCNQ1	1.11	G/A	1.02	0.70
	(rs231361)^#^			0.51	(0.94–1.10)	

* Risk/non-risk allele and risk allele frequency in Europeans

~ Covariates used in PanScan datasets include age, sex, study site, genotypic race (Eigenstrat principal components 1 and 2), and other significant principal components.

# Variant within the bracket is within high LD of the targeted SNP and is used to represent the association between the targeted variant and pancreatic cancer risk

^f^ Association in females

^m^ Association in males

In the combined data, four SNPs were associated with PaC risk. These included two variants, *FTO* rs8050136 (OR = 1.08, 95% CI 1.01–1.16, p = 0.03) and *BCL11A* rs243021 (OR = 0.90, 95% CI 0.84–0.97, p = 0.005), which confirmed the reported results of the PanScan 1 data (same direction and similar effect size) [12]. The other two variants were newly identified as *JAZF1* rs1635852 (OR = 0.9, 95% CI 0.84–0.96, p = 0.003) and *GCK* rs4607517 (OR = 1.11, 95% CI 1.01–1.21, p = 0.03). In the PanScan 2 dataset (data not shown), the three SNPs (rs1387153, rs243021, and rs8050136) previously identified in PanScan 1 [12] did not replicate.

After stratifying by diabetes status in the Mayo Clinic subset, we detected five significant associations at p = 0.05 level. Focusing on long-standing diabetic PaC cases and controls, we identified *SLC30A8* rs13266634 (OR = 4.92, 95% CI 1.71–14.2, p = 0.003). Focusing on PaC cases and controls without diabetes, we identified *DGKB-TMEM195* rs2191348 (OR = 1.23, 95% CI 1.03–1.47, p = 0.02), *KCNQ1* rs231362 (OR = 1.24, 95% CI 1.00–1.53, p = 0.046), *ADCY5* rs2877716 (OR = 1.28, 95% CI 1.05–1.58, p = 0.02) and *BCL11A* rs243021 (OR = 0.83, 95% CI 0.69–0.99, p = 0.04) (Table 2).

**Table 2:** Association analysis of Type 2 diabetes mellitus (T2DM) susceptibility variants and pancreatic cancer risk stratified by diabetes status using the Mayo Clinic subset.

Chromosome	SNP	Gene Region	T2DM Odds Ratio	Risk/non-risk allele Risk allele frequency*	Long-standing Diabetes (63 cases and 23 controls)			No Diabetes (448 cases and 557 controls)		
					Odds Ratio	95% C.I.	p	Odds Ratio	95% C.I.	p
**SNPS observed in GWAS**										
1	rs2641348	NOTCH2	1.13	C/T	0.92	0.40–2.12	0.85	1.04	0.79–1.37	0.79
				0.11						
1	rs340874	PROX1	1.07	G/A	1.35	0.60–3.02	0.47	1.05	0.88–1.26	0.56
				0.54						
2	rs13414140	THADA	1.15	C/T	0.43	0.10–1.76	0.24	0.89	0.68–1.15	0.37
				0.91						
2	rs243021	BCL11A	1.08	T/C	0.75	0.33–1.70	0.49	0.83	0.69–0.99	0.04
				0.46						
2	rs2943641	IRS1	1.11	C/T	1.54	0.63–3.77	0.34	1.10	0.91–1.32	0.31
				0.63						
2	rs780094	GCKR	1.06	G/A	1.44	0.70–2.97	0.33	1.02	0.84–1.22	0.87
				0.59						
3	rs2877716	ADCY5	1.12	C/T	0.79	0.30–2.10	0.64	1.28	1.05–1.58	0.02
				0.81						
3	rs4402960	IGF2BP2	1.14	T/G	1.56	0.63–3.86	0.34	1.03	0.85–1.24	0.80
				0.29						
3	rs4411878	ADAMTS9	1.08	C/T	1.02	0.44–2.35	0.97	0.89	0.72–1.10	0.28
				0.79						
3	rs6802898	PPARG	1.14	C/T	1.04	0.28–3.90	0.95	1.01	0.77–1.32	0.95
				0.88						
4	rs10012946	WFS1	1.12	C/T	0.44	0.18–1.04	0.06	0.91	0.76–1.09	0.31
				0.63						
5	rs7708285	ZEBD3	1.08	G/A	1.42	0.62–3.26	0.41	1.06	0.87–1.30	0.54
				0.31						
6	rs7756992	CDKAL1	1.14	G/A	1.62	0.68–3.83	0.28	0.96	0.78–1.19	0.73
				0.27						
7	rs13234407	KLF14	1.07	G/A	0.82	0.36–1.85	0.63	1.10	0.92–1.32	0.28
				0.54						
7	rs1635852	JAZF1	1.1	T/C	0.64	0.28–1.46	0.28	0.99	0.83–1.19	0.91
				0.46						
7	rs2191348	DGKB-	1.06	T/G	2.15	0.97–4.78	0.06	1.23	1.03–1.47	0.02
		TMEM195		0.52						
7	rs4607517	GCK	1.07	A/G	1.55	0.55–4.43	0.41	1.17	0.92–1.48	0.20
				0.18						
8	rs13266634	SLC30A8	1.15	C/T	4.92	1.71–14.2	0.003	0.91	0.75–1.10	0.32
				0.71						
8	rs896854	TP53IMP1	1.06	A/G	0.87	0.40–1.91	0.73	0.90	0.75–1.07	0.22
				0.46						
9	rs10512085	CHCHD9	1.11	A/G	3.20	0.76–13.4	0.11	1.30	0.92–1.84	0.14
				0.92						
9	rs2383208	CDKN2A/B	1.20	A/G	0.90	0.29–2.83	0.86	0.93	0.74–1.16	0.51
				0.83						
10	rs1111875	HHEX/IDE	1.15	G/A	0.41	0.15–1.12	0.08	0.94	0.79–1.12	0.51
				0.55						
10	rs11257655	CDC123	1.11	T/C	1.27	0.47–3.42	0.63	0.94	0.76–1.16	0.57
				0.24						
10	rs7903146	TCF7L2	1.37	T/C	0.88	0.41–1.89	0.75	1.06	0.86–1.29	0.60
				0.31						
11	rs1387153	MTNR1B	1.15	T/C	2.40	0.95–6.07	0.06	1.09	0.90–1.32	0.38
				0.28						
11	rs1552224	CENTD2	1.14	T/G	0.77	0.27–2.17	0.62	1.01	0.79–1.29	0.94
				0.84						
11	rs2237892	KCNQ1	1.29	C/T	0.91	0.15–5.41	0.92	1.01	0.72–1.42	0.96
				0.93						
11	rs2334499	H19/IGF2	1.08	T/C	1.23	0.52–2.86	0.64	1.13	0.94–1.37	0.19
				0.43						
11	rs5215	KCNJ11	1.14	C/T	0.82	0.36–1.86	0.63	1.09	0.90–1.32	0.38
				0.34						
12	rs1353362	TSPAN8	1.09	C/T	1.24	0.51–3.05	0.63	0.90	0.74–1.10	0.31
				0.29						
12	rs2612067	HMGA2	1.10	C/A	0.70	0.27–1.84	0.47	1.21	0.90–1.62	0.20
				0.12						
12	rs7965349	HNF1A	1.07	C/T	1.25	0.50–3.07	0.63	0.91	0.73–1.13	0.39
				0.81						
15	rs4778582	ZFAND6	1.06	A/G	0.69	0.26–1.78	0.44	0.98	0.80–1.19	0.82
				0.67						
15	rs8042680	PRC1	1.07	A/C	0.97	0.48–1.99	0.94	1.04	0.86–1.25	0.68
				0.34						
16	rs8050136	FTO	1.17	A/C	1.33	0.61–2.88	0.47	1.05	0.87–1.26	0.64
				0.42						
17	rs4430796	HNF1B	1.10	G/A	0.75	0.35–1.63	0.47	1.01	0.85–1.21	0.90
				0.47						
X	rs5945326^f^	DUSP9	1.27	A/G	1.18	0.31–4.41	0.81	1.10	0.80–1.51	0.56
				0.74						
X	rs5945326^m^	DUSP9	1.27	A/G	0.50	0.08–3.08	0.45	1.09	0.73–1.62	0.69
				0.74						
**SNPs in high LD with susceptibility variants**										
11	rs231362^#^	KCNQ1	1.11	G/A	1.00	0.39–2.62	0.99	1.24	1.00–1.53	0.046
	(rs231361)			0.51						

* Risk/non-risk allele and risk allele frequency in Europeans

# Variant within the bracket is within high LD of the targeted SNP and is used to represent the association between the targeted variant and pancreatic cancer risk

^f^ Association in females

^m^ Association in males

NA: not available

Overall, four associations in PanScan sets reached statistical significance at a threshold of p = 0.05. However, none of them were significant after correction for multiple comparisons using the Bonferroni correction and FDR correction.

### Association with type 2 diabetes

Among the 14 reported PaC susceptibility variants, 11 were genotyped in the GENEVA diabetes GWAS (n = 5) or were represented by SNPs in high LD (n = 6). Variants *ABO* rs505922, *PDX1* rs9581943 and *TERT* rs2736098 were not captured in these datasets. Only the association between *LINC-PINT* rs6971499 and T2DM risk appeared significant at p = 0.05 level in the combined dataset of NHS and HPFS, however, it was no longer significant after correction for multiple comparisons (Table 3).

**Table 3:** Association between pancreatic cancer susceptibility variants with Type 2 diabetes (T2DM) risk in the GENEVA study.

Chromosome	SNP	Gene Region	Pancreatic Cancer Odds Ratio (95% C.I.)	Minor/Reference allele, MAF*	NHS dataset^~^(1579 cases; 1801 controls)		HPFS dataset^~^ (1162 cases; 1336 controls)		Combined dataset (2741 cases; 3137 controls)	
					Odds Ratio (95% C.I.)	p	Odds Ratio (95% C.I.)	p	Odds Ratio (95% C.I.)	p
**SNPs observed in GWAS**										
1	rs12029406	NR5A2	0.83 (0.78–0.89)	T/C	0.99	0.83	1.06	0.38	1.02	0.62
				0.43	(0.88–1.11)		(0.93–1.21)		(0.94–1.11)	
1	rs3790843	NR5A2	0.81 (0.75–0.87)	T/C	0.95	0.40	1.09	0.25	1.01	0.86
				0.31	(0.84–1.07)		(0.95–1.25)		(0.92–1.11)	
1	rs3790844	NR5A2	0.77 (0.71–0.84)	G/A	1.03	0.70	1.08	0.29	1.06	0.27
				0.26	(0.90–1.18)		(0.94–1.25)		(0.96–1.17)	
5	rs401681	CLPTM1L-	1.19 (1.11–1.27)	T/C	0.99	0.82	1.06	0.36	1.03	0.55
		TERT		0.46	(0.88–1.11)		(0.93–1.21)		(0.94–1.12)	
16	rs7190458	BCAR1/CTRB1/	1.46 (1.30–1.65)	A/G	1.27	0.10	0.86	0.32	1.08	0.46
		CTRB2		0.04	(0.95–1.69)		(0.63–1.16)		(0.88–1.32)	
**SNPs in high LD with susceptibility variants**										
1	rs10919791	NR5A2	0.77 (0.71–0.84)	A/G	1.11	0.26	1.02	0.85	1.05	0.28
	(SNP_A-			0.24	(0.92–1.34)		(0.85–1.22)		(0.96–1.14)	
	4218585,									
	SNP_A-									
	8512892)^#^									
1	rs4465241	NR5A2	1.25 (1.14–1.37)	T/C	1.11	0.26	1.02	0.85	1.05	0.28
	(SNP_A-			0.18	(0.92–1.34)		(0.85–1.22)		(0.96–1.14)	
	4218585,									
	SNP_A-									
	8512892)^#^									
13	rs9543325	near FABP5L1	1.26 (1.18–1.35)	C/T	1.48	0.18	1.07	0.80	1.20	0.35
	(SNP_A-			0.39	(0.84–2.62)		(0.63–1.82)		(0.82–1.76)	
	8564737) ^#^									
13	rs9564966	near FABP5L1	1.21 (1.13–1.30)	A/G	1.44	0.23	0.80	0.44	0.96	0.45
	(SNP_A-			0.34	(0.79–2.62)		(0.45–1.42)		(0.88–1.06)	
	1804006,									
	SNP_A-									
	2157058) ^#^									
7	rs6971499	LINC-PINT	0.79 (0.74–0.84)	C/T	0.86	0.06	0.93	0.45	0.88	0.04
	(SNP_A-			0.12	(0.73–1.01)		(0.78–1.12)		(0.79–0.99)	
	8625603) ^#^									
22	rs16986825	ZNRF3	1.18 (1.12–1.25)	T/C	0.95	0.56	1.11	0.23	1.03	0.61
	(SNP_A-			0.16	(0.82–1.12)		(0.93–1.33)		(0.92–1.16)	
	1800206) ^#^									

* Minor/Reference allele, minor allele frequency (MAF) in Europeans

# Variants within the bracket are within LD of the targeted SNP and are used to represent the association between the targeted variant and T2DM risk

~ NHS denotes Nurses' Health Study; HPFS denotes Health Professionals Follow-up Study

## Discussion

Based on previous results that demonstrated a relationship between T2DM and PaC, we hypothesized a shared genetic etiology. Using published GWAS data, we tested the association between reported T2DM susceptibility variants and PaC risk, as well as the association between reported PaC susceptibility variants and T2DM risk. The analyses showed that only one PaC susceptibility variant was associated with T2DM risk at a weak significance level; similarly, there were only weak associations between T2DM susceptibility variants and PaC risk. These associations, found at a significance threshold of p = 0.05 became not significant after adjustment for multiple comparisons. We also did not replicate any of the three T2DM SNPs from the PanScan 1 dataset analysis in the PanScan 2 dataset.

One design strength of our study is that we could perform initial stratified analyses by diabetes status of PaC cases and controls to evaluate whether associations between T2DM susceptibility variants and PaC risk differ by diabetes history. We found no associations significant, despite one potential association showing a relatively large effect size (rs13266634, OR = 4.92). We discounted the large effect size for rs13266634 because it was likely due to the subgroup sample sizes of 63 cases and 23 controls, and the frequency of the minor allele (T) in the control subjects was not congruent with reports in the general population (0.46 vs 0.29). The sample size of the PaC cases and controls without diabetes (448 cases and 557 controls), was likely underpowered as well, but our findings on *DGKB-TMEM195* rs2191348, *KCNQ1* rs231362, *ADCY5* rs2877716 and *BCL11A* rs243021 may warrant further evaluation from larger studies.

Our study has other limitations. We did not evaluate susceptibility variants from candidate gene association studies. Many biologically plausible candidate genes have been reported to be associated with T2DM and PaC in different studies [29,30]. However, it is well known that the replication rate for SNPs derived using the candidate gene association design is relatively low [6]. Thus rather than test variants which were not always demonstrated to be associated with disease risk, we focused on GWAS derived susceptibility SNPs which were consistently replicated.

One possible explanation for these results is that the variants we analyzed from the GWAS studies account for only a modest genetic susceptibility to disease risk. There may still be genetic loci beyond these GWAS-derived SNPs that could play a role that link these two diseases. It is also likely that the majority of the shared etiology of PaC and T2DM involve other factors beyond genetic level, such as obesity, epigenetic or environmental factors. Shared family environment could potentially explain the observed association between family history of diabetes and pancreatic cancer risk. Complementary genetic methods, such as family-based linkage studies or high throughput sequencing studies, may offer alternatives to characterize the potential shared genetic etiology of the two conditions, and may reveal novel associations or interactions.

In conclusion, we found that GWAS-derived susceptibility variants do not explain the potential shared genetic etiology of PaC and T2DM. We do report interesting associations that may warrant further study using independent datasets with larger sample sizes.
